# Obstructive Sleep Apnea-Hypopnea Syndrome in Children With Down Syndrome Treated at a Tertiary Care Hospital in Northeastern Colombia

**DOI:** 10.7759/cureus.102624

**Published:** 2026-01-30

**Authors:** Silvia N Suarez Mantilla, Victor Manuel Mora Bautista, Martha Lucia Africano Leon, Diana C Vergara Arenas, Yuli E Rojas, Sergio Serrano-Gomez

**Affiliations:** 1 Pediatrics, Universidad Industrial de Santander, Bucaramanga, COL; 2 Pediatrics, Clinica Materno Infantil San Luis, Bucaramanga, COL; 3 Paediatrics and Child Health, Universidad Industrial de Santander, Bucaramanga, COL

**Keywords:** colombia, down syndrome, obstructive sleep apnea syndrome, pediatrics & child health, polysomnography, polysomnography (psg)

## Abstract

Obstructive sleep apnea-hypopnea syndrome (OSAHS) is frequent in children with Down Syndrome (DS), associated with anatomical characteristics such as midfacial hypoplasia, macroglossia, and hypotonia. It represents a frequent cause of morbidity and mortality among children with DS. Early identification of OSAHS in the DS population is essential, as untreated sleep breathing disorders are linked to severe complications, including pulmonary hypertension. Consequently, guidelines, such as the *Health Supervision for Children with Down Syndrome *from the American Academy of Pediatrics, recommend polysomnography (PSG) for all children with DS before age 4. This case-control study examined the profile of OSAHS in 124 patients with DS under 13 years of age who were treated at a tertiary care institution in northeastern Colombia from 2012 to 2023. Of the 124 patients, 19 (15.3%) were diagnosed with OSAHS. The only significant association identified was with place of residence, related to the fact that individuals (not patients) living in the city or its metropolitan area have greater access to PSG. Our observed frequency is lower than previously reported in the literature, indicating possible systematic underdiagnosis. Socioeconomic status and access to diagnostic evaluation, rather than clinical characteristics, appear to be the primary determinants for receiving a diagnosis. Expanding access to PSG, particularly in rural and low-income regions, and prioritizing universal screening in clinical protocols over symptom-based referral are essential steps.

## Introduction

Down syndrome (DS) is the most common chromosomal abnormality and is caused by an extra copy of chromosome 21 [1]. Worldwide, it affects about 1 in every 600 to 800 live births [2-6]. In Mexico, the rate is 3.73 per 10,000 live births [7], while in Colombia it is 1.72 per 1,000 live births (or 7-9 per 10,000), though there are regional differences and recent national data are lacking [8-11]. People with DS have distinctive physical traits, intellectual disability, and a higher risk of other health problems. Thanks to medical advances, especially in treating congenital heart disease, life expectancy for people with DS is now close to 60 years. Still, respiratory diseases are a major cause of illness and death [2,11].

Sleep-related breathing disorders (SBD) are very common in children with DS, but they are often missed or not diagnosed [2,3]. The most serious of these is obstructive sleep apnea-hypopnea syndrome (OSAHS), which affects between 31% and 79% of children with DS [2,3]. In comparison, only about 2-4% of the general population is affected [2,12]. In Colombia, up to 82% of children with DS have SBD, while the rate in the general population is just 0.02% [12,13].

The elevated OSAHS prevalence in children with DS stems primarily from anatomical and functional characteristics inherent to the syndrome [14]. These include midfacial and mandibular hypoplasia, upper airway narrowing, adenotonsillar hypertrophy, macroglossia, and generalized muscular hypotonia that affects the pharyngeal musculature [15]. Additionally, the increased risk of obesity further contributes to susceptibility [16]. Collectively, these features compromise airway patency and elevate the likelihood of collapse during inspiration [2,3].

It is important to identify OSAHS early in children with DS because untreated sleep-breathing problems can lead to serious issues like worsening pulmonary hypertension, right-sided heart failure, severe shortness of breath, and pulmonary arterial hypertension [3,12,16]. Since there is often a weak link between what caregivers notice and what tests show, polysomnography (PSG) is considered the best way to confirm and measure OSAHS. It is recommended for all children with DS by age 4 [14]. In Colombia, a lack of data makes it hard to develop diagnostic and treatment plans that fit the country’s needs [3,12].

## Materials and methods

Objective

Analyze the factors associated with OSAHS in pediatric patients with DS treated at a tertiary hospital in Bucaramanga, Colombia.

Study design

We conducted an unmatched retrospective case-control study. We used electronic medical records of patients younger than 13 years with confirmed DS who visited Clínica Materno Infantil San Luis in Bucaramanga, Colombia. We compared exposures and clinical characteristics between cases (OSAHS-positive) and controls (OSAHS-negative) to identify factors associated with OSAHS from January 1, 2012, to December 31, 2023.

The research was carried out between January and December 2024.

Study population

We reviewed electronic medical records for patients with DS. Cases were defined as patients diagnosed with obstructive sleep apnea by PSG, as documented in clinical records. Controls were patients without this diagnosis. Controls were selected from the same population and time period as cases to minimize selection bias. Both groups were chosen using simple random sampling. We included participants diagnosed with DS who were younger than 13 at the time of consultation and had attended at least one medical follow-up during the study period. Those with missing or incomplete medical records were excluded. Central sleep apnea events were not included in the analysis.

Data collection and analysis

Electronic medical records were reviewed to identify eligible subjects using the following ICD-10 codes: Q900, Q901, Q902, and Q909 [17]. Among patients with DS who underwent PSG during the study period, 19 cases with OSAHS and 105 controls without OSAHS were identified, totaling 124 patients included in the analysis. Data were obtained exclusively from the hospital's internal electronic medical record system. Each record was assigned a unique code and contained no personally identifiable information. Data were stored in a password-protected database.

Obstructive sleep apnea-hypopnea syndrome definition

Diagnosis was established using PSG and defined by an apnea-hypopnea index (AHI) of one or more events per hour [18]. The AHI quantifies the frequency of respiratory disturbances during sleep and is used to assess disease severity and inform treatment decisions [18].

Statistical analysis

We used measures of central tendency and dispersion to describe quantitative variables. We reported absolute and relative frequencies for qualitative variables. To analyze the association between categorical variables and OSAHS, we used Fisher's exact test for 2×2 tables when expected cell frequencies were <5, and the Chi-square test for larger tables and when all expected cell frequencies were ≥5.. We calculated odds ratios (OR) with 95% confidence intervals to estimate the strength of associations between exposures and OSAHS. Percentages were calculated excluding cases with unknown values. All statistical tests were two-sided. P-values <0.05 were considered statistically significant. All analyses were performed using STATA version 16 (StataCorp., College Station, TX).

Ethical aspects

This study was reviewed and approved by the ethics committee of Clinica Materno Infantil San Luis (approval no. 23072024). The research followed the principles of the Declaration of Helsinki, the Guidelines of the Council for International Organizations of the Medical Sciences, and Resolution 008430 of October 4, 1993, from the Ministry of Health of Colombia. Since this study involved only the review of electronic medical records without direct patient involvement, it was classified as minimal risk research. Informed consent from participants was not required [19-21].

## Results

The study included 124 patients with DS, of whom 19 (15.3%) had OSAHS. Most participants were male (74, 59.7%), born at term (81, 65.3%), and delivered by cesarean section (75, 60.5%). The mean maternal age was 34.8 years, and 55 (44.1%) mothers were 35 years or older at delivery. DS was diagnosed prenatally in 74 (59.7%) patients. Regarding birth weight, 42 (33.9%) patients had low birth weight (<2,500 g), while 73 (58.9%) had normal birth weight (2,500-3,999 g). Nutritional assessment showed that 45 (36.3%) patients were undernourished, 57 (46.0%) had normal nutritional status, and 5 (4.0%) were overweight. Most patients, 82 (66.1%), resided in the metropolitan area, including Bucaramanga, Girón, and Floridablanca. Regarding health insurance, 68 (54.8%) patients were enrolled in the contributory system (which requires a financial contribution from the patient).

No statistically significant associations were found between OSAHS and the following variables: sex (male: 13.5% vs. female: 18.0%; *P *= 0.613), maternal age (<35 years: 19.1% vs. ≥35 years: 7.3%; *P* = 0.083), gestational age at birth (χ² (3) = 1.75, *P* = 0.628), mode of delivery (cesarean: 18.7% vs. vaginal: 10.2%; χ²(1) = 1.04, *P* = 0.308), health insurance type (χ²(2) = 5.08, *P* = 0.079), timing of DS diagnosis (prenatal: 20.3% vs. postnatal: 8.5%; χ²(1) = 2.37, *P* = 0.123), or nutritional status (*P* = 0.401). Regarding birth weight (low birth weight: 26.2% vs. normal weight: 11.0%; *P* = 0.114), although the odds ratio (OR) suggests a clinically meaningful association, statistical significance was likely not reached due to insufficient sample size. A statistically significant difference by residential area was identified (χ²(1) = 11.53, *P* = 0.002). OSAHS was more common in metropolitan than rural patients (18, 22.0%, vs. 1, 2.4%). This is a ninefold difference across locations. Of 82 metropolitan patients, 18 (22.0%) had OSAHS; only 1 of 42 rural patients (2.4%) had a documented diagnosis.

In multivariate logistic regression analysis, metropolitan residential area remained an independent risk factor for OSAHS (adjusted OR = 10.85; 95% confidence interval (CI): 1.35-87.23; *P* = 0.025) after adjusting for birth weight. Low birth weight (<2,500 g) showed a trend toward association that did not reach statistical significance (adjusted OR = 2.45; 95% CI: 0.87-6.91; *P* = 0.089) (Table 1).

**Table 1:** Characteristics associated with OSAHS. Percentages were calculated based on valid cases (excluding unknown values). *P*-values were calculated using Fisher’s exact test for 2 × 2 tables when expected cell frequencies were <5 and the chi-square test for larger contingency tables or when all expected cell frequencies were ≥5. *vs. ≥37 weeks. **vs. subsidized insurance. ***vs. normal/healthy χ², chi-square statistics; df, degrees of freedom; OR, odds ratio; CI, confidence interval; OSAHS, obstructive sleep apnea-hypopnea syndrome

Variable	Category	Total (*n* = 124)	No OSAHS (*n* = 105)	%	OSAHS (*n* = 19)	%	*P*-value	OR	95% CI
Sex							0.613	0.71	0.27-1.87
	Male	74	64	86.5	10	13.5			
	Female	50	41	82.0	9	18.0			
Maternal age							0.083	2.96	0.87-10.1
	<35 years	47	39	80.9	9	19.1			
	≥35 years	55	51	92.7	4	7.3			
	Unknown	22	16		6	27.2			
Gestational age							0.628		
	<28 weeks	2	2	100	0	0.0			
	28-31.6 weeks	4	3	75.0	1	25.0		1.60*	0.15-16.45
	32-33.6 weeks	35	31	88.6	4	11.4		0.62*	0.19-2.03
	≥37 weeks	81	67	82.7	14	17.3			
	Unknown	2	2		0				
Birth weight									
	<2,500 g	42	31	73.8	11	26.2		2.88	1.05-7.89
	2,500-3,999 g	73	65	89.0	8	11.0			
	≥4,000 g	3	3	100	0	0.0			
	Unknown	6	6		0				
Mode of delivery							0.308	2.01	0.69-5.87
	Cesarean section	75	61	81.3	14	18.7			
	Vaginal	49	44	89.8	5	10.2			
Health insurance type							0.079		
	Contributory	68	54	79.4	14	20.6		2.85**	0.88-9.28
	Subsidized	48	44	91.7	4	8.3		1.57	0.15-16.13
	Special regime	8	7	87.5	1	12.5			
Residential area							0.002	11.53	1.48–89.68
	Metropolitan	82	64	78.0	18	22.0			
	Rural	42	41	97.6	1	2.4			
Timing of diagnosis							0.123	2.67	0.83-8.60
	Prenatal	74	59	79.7	15	20.3			
	Postnatal	47	43	91.5	4	8.5			
	Unknown	3	3		0				
Nutritional status									
	Undernutrition	45	35	77.8	10	22.2	0.401	1.75***	0.63-4.88
	Normal/Healthy	57	49	86.0	8	14.0			
	Overweight/Obesity	5	5	100	0	0.0			
	Unknown	17	16		1				
Total		124	105	84.7	19	15.3			

## Discussion

This study found that children with DS living in metropolitan areas had a significantly higher OSAHS diagnosis rate than those in rural regions (18, 22.0%, vs 1, 2.4%, *P *= 0.002). This ninefold disparity is more likely attributable to detection bias than to an actual difference in disease prevalence.

Children from higher-income or metropolitan areas have substantially greater access to specialized healthcare services, including pediatric sleep medicine and PSG facilities, than those from lower-income regions. This disparity highlights a significant gap in equitable healthcare delivery for children with DS in Colombia, especially considering that guidelines, such as Health Supervision for children and adolescents with Down syndrome, recommend PSG by age four for all children with DS [22]. The urgency of this issue is amplified by the severity of OSAHS in the DS population and the necessity of early diagnosis to prevent serious complications, such as pulmonary hypertension, right-sided heart failure, and neurocognitive impairment [3,23].

Several factors contribute to the observed socioeconomic disparities in OSAHS diagnosis through PSG access. Pediatric sleep laboratories are predominantly located in cities with tertiary care centers, creating geographic barriers such as long travel distances, inadequate infrastructure, and limited transportation options for families from disadvantaged regions. Furthermore, the financial burden, including both direct out-of-pocket costs and indirect expenses such as transportation, lost wages, and overnight accommodations, further limits access for lower-income households. Addressing these barriers will require comprehensive strategies, including targeted public policy interventions, infrastructure investment, community outreach, family education, and innovative care models to promote equitable access for rural populations [24-27].

The absence of other significant associations suggests that access to diagnostic procedures, rather than clinical profile, is currently the primary determinant of which children receive PSG in this population. This pattern suggests systematic underdiagnosis of OSAHS among children with DS from low-income settings, with potential adverse consequences for long-term health and development.

Implications and recommendations

These findings have significant implications for public health policy and clinical practice in Colombia. Expanding PSG access in underserved regions is urgently needed and may be achieved through innovative delivery models, such as mobile sleep laboratories, validated, simplified home sleep testing protocols adapted for pediatric DS populations, increased capacity at regional hospitals, or other strategies for diagnosis.

Additionally, healthcare systems should implement proactive case-finding strategies in lower-income areas through systematic screening programs that do not depend solely on parental symptom recognition or spontaneous specialist referrals.

Given the well-established poor correlation between parental-reported symptoms and PSG findings in DS [12], clinical protocols should prioritize universal screening over symptom-based referrals, especially in underserved populations where healthcare literacy barriers and diagnostic delays are more prevalent. Universal screening aligns with international best practices and addresses the diagnostic challenges specific to this population.

Furthermore, health insurance policies should ensure that PSG is fully covered for children with DS as a preventive service, thereby eliminating financial barriers, and implementing a national guide adapted to our economic, social, and geographical conditions is essential to this essential diagnostic evaluation and promoting equitable access regardless of socioeconomic status or geographic location [27].

Limitations

Our study has several limitations. First, it is a retrospective review of medical records from a single tertiary center (Clínica Materno Infantil San Luis); therefore, the findings may not be generalizable to all children with Down syndrome in Bucaramanga or Colombia. This design also limited the ability to control for potential confounders, including access to healthcare, healthcare provider, parental education level, clinical severity of DS, and comorbidities. Selection bias is present because only children who received care at this tertiary hospital were included. Consequently, the number of children with undiagnosed OSAHS who never reached tertiary care remains unknown and is likely substantial. In addition, the study cannot fully differentiate the true burden of OSAHS from detection bias. Children from rural areas may have undiagnosed OSAHS due to limited access to PSG. Furthermore, the absence of PSG in most controls further limits comparability between cases and controls. Because PSG was primarily performed in patients with higher clinical suspicion, controls with undiagnosed OSAHS may have been misclassified. PSG results were evaluated regardless of the performing center, which limited access to detailed information such as symptom severity, polysomnographic parameters, equipment used, scoring personnel, laboratory standards, and treatment outcomes. Finally, classifying socioeconomic status by place of residence, while informative, may not capture the full spectrum of economic factors influencing healthcare access.

## Conclusions

Where a child lives, which is often linked to their family's income and access to healthcare, plays a significant role in the likelihood of receiving an OSAHS diagnosis, independent of clinical symptomatology. Rather than indicating differences in disease risk, these results point to important disparities in access to diagnostic evaluation, particularly polysomnography. It is essential to improve access to PSG in rural and low-income areas and make universal screening a routine part of care rather than relying solely on referrals based on symptoms. These results show that there are major gaps in healthcare for diagnosing OSAHS in children with Down syndrome in Colombia and underscore the urgent need for targeted actions to ensure equal access to essential diagnostic tests for all children.
